# Supplementary material for: Nosocomial Outbreak of Ralstonia pickettii Infections Likely Linked to Saline Solutions in Germany from August 2023 to March 2024—Challenges in Medical Product-Related Outbreaks
Source: Microorganisms. 2025 Sep 9;13(9):2102. doi: 10.3390/microorganisms13092102 (PMC12472745; doi:10.3390/microorganisms13092102)
Supplement: Supplementary file 1 [file microorganisms-13-02102-s001.zip › microorganisms-3810530-supplementary.pdf]

Supplementary Material

For manuscript titled “Nosocomial outbreak of *Ralstonia pickettii* infections likely linked to saline solutions in Germany from August 2023 until March 2024 – Challenges in medical product-related outbreaks”

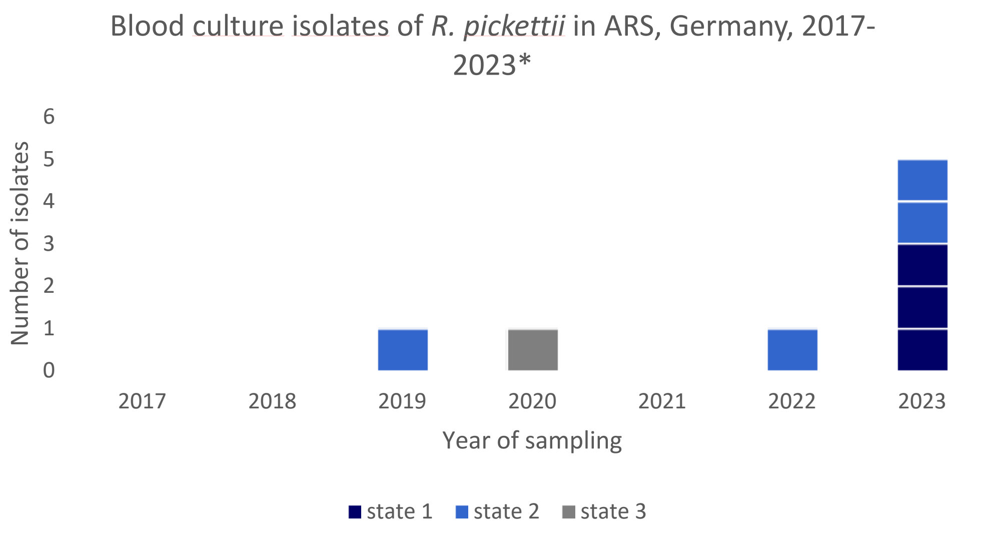

Figure S1. Blood culture isolates of *Ralstonia pickettii* in the Antibiotic Resistance Surveillance (ARS) system in Germany, among continuously participating hospitals, 2017-2023. *Data retrieved on 12/2023

Table S1. Metadata for the 28 *R. pickettii* samples included in this study. An asterisk in the coverage column indicates values taken from NCBI. For hybrid assemblies, coverage is shown as long-read + short-read (ONT + Illumina). Illumina coverage was calculated using bbmap (via GARI), Flye coverage was derived from the assembler itself, and Unicycler coverage was estimated based on number of base-pairs sequenced divided by the genome size. Secondary genomic species determination according to the Genome Taxonomy Database (GTDB, https://gtdb.ecogenomic.org) and the Type Strain Genome Server (TYGS, https://tygs.dsmz.de) assigned isolates from some excluded cases to several distinct *Ralstonia* species as documented in the respective columns.

ID	Country	State	Location	Collection_ Year	Seq_Strategy	Assembly_Method	Study	Case_ classification	Coverage	GTDB_ species	TYGS_ Species
**HY4163**	Germany	State 2	Hospital 01	2023	Short Read	Velvet (v1.1.04)	Krone et al., 2024		59*	*R. thomasii*	*R. thomasii*
**HY4145**	Germany	State 2	Hospital 01	2023	Short Read	Velvet (v1.1.04)	Krone et al., 2024		51*	*R. pickettii*	*R. pickettii*
**HY4115**	Germany	State 2	Hospital 01	2023	Short Read	Velvet (v1.1.04)	Krone et al., 2024	confirmed	50*	*R. pickettii*	*R. pickettii*
**HY4146**	Germany	State 2	Hospital 01	2023	Short Read	Velvet (v1.1.04)	Krone et al., 2024	confirmed	47*	*R. pickettii*	*R. pickettii*
**HY4149**	Germany	State 2	Hospital 01	2023	Short Read	Velvet (v1.1.04)	Krone et al., 2024	confirmed	27*	*R. pickettii*	*R. pickettii*
**OR2283**	Germany	State 2	Hospital 01	2024	Long Read + Short Read	Unicycler (v0.5.0)	This study	confirmed	1.78 + 107.77	*R. pickettii*	*R. pickettii*
**23_B33984**	Germany	State 7	Hospital 02	2023	Long Read + Short Read	Flye (v2.9.3) + Polypolish (v0.6.0)	This study	confirmed	191 + 67.31	*R. pickettii*	*R. pickettii*
**1728446771**	Germany	State 4	Hospital 03	2024	Short Read	SPAdes (v3.15.5)	This study	confirmed	37.22	*R. pickettii*	*R. pickettii*
**HY4261**	Germany	State 2	Hospital 01	2024	Short Read	SPAdes (v3.15.5)	This study	excluded	130.02	*R. pickettii*	*R. pickettii*
**BK115556**	Germany	State 3	Hospital 04	2024	Long Read + Short Read	Flye (v2.9.3) + Polypolish (v0.6.0)	This study	excluded	198 + 83.51	*R. pickettii*	*R. pickettii*
**BB_XA27_0046**	Germany	State 5	Hospital 05	2024	Long Read + Short Read	Flye (v2.9.3) + Polypolish (v0.6.0)	This study	excluded	200 + 51.94	*R. thomasii*	*R. thomasii*
**BB_XB07_0060**	Germany	State 5	Hospital 05	2024	Long Read + Short Read	Flye (v2.9.3) + Polypolish (v0.6.0)	This study	excluded	198 + 15.46	*R. thomasii*	*R. thomasii*
**302729**	Germany	State 8	Hospital 06	2024	Short Read	SPAdes (v3.15.5)	This study	excluded	143.24	*R. pickettii*	*R. pickettii*
**1042061218**	Germany	State 7	Hospital 07	2023	Short Read	SPAdes (v3.15.5)	This study	excluded	45.64	*R. thomasii*	*R. thomasii*
**9868178**	Germany	State 6	Hospital 08	2024	Short Read	SPAdes (v3.15.5)	This study	excluded	40.46	*R. thomasii*	*R. thomasii*
**KH-09685**	Germany	State 6	Hospital 09	2024	Short Read	SPAdes (v3.15.5)	This study	excluded	94.82	inconclusive	inconclusive
**KH-09780-1000**	Germany	State 6	Hospital 09	2024	Short Read	SPAdes (v3.15.5)	This study	excluded	90.01	inconclusive	inconclusive
**UC7445**	Germany	State 1	Hospital 10	2024	Short Read	SPAdes (v3.15.5)	This study	excluded	23.91	*R. thomasii*	*R. thomasii*
**VA408617**	Germany	State 2	Hospital 11	2024	Short Read	SPAdes (v3.15.5)	This study	excluded	77.02	inconclusive	inconclusive
**VA408619**	Germany	State 2	Hospital 11	2024	Short Read	SPAdes (v3.15.5)	This study	excluded	78.58	inconclusive	inconclusive
**SRR11285235**	Netherlands	Netherlands	Netherlands	2003	Short Read	SPAdes (v3.15.5)	Fluit et al., 2021		62.14	*R. pickettii*	*R. pickettii*
**SRR11285240**	Netherlands	Netherlands	Netherlands	2012	Short Read	SPAdes (v3.15.5)	Fluit et al., 2021		49.99	*R. pickettii*	*R. pickettii*
**SRR11285251**	Netherlands	Netherlands	Netherlands	2012	Short Read	SPAdes (v3.15.5)	Fluit et al., 2021		76.17	*R. pickettii*	*R. pickettii*
**SAMN38255698**	Australia	Australia	Australia	2023	Short Read	SKESA (v2.3.0)	BioProject PRJNA1040313		63*	*R. pickettii*	*R. pickettii*
**SAMN08239918**	China	China	China	2016	Short Read	SPAdes (v3.0.0)	Zheng et al., 2017		150*	inconclusive	*R. flatus*
**SAMEA115737708**	UK	UK	UK	2023	Short Read	SPAdes (v3.15.5)	Saunders et al., 2024		41.24	*R. pickettii*	*R. pickettii*
**SAMEA115737709**	UK	UK	UK	2023	Short Read	SPAdes (v3.15.5)	Saunders et al., 2024		32.51	*R. pickettii*	*R. pickettii*
**SAMEA115743540**	UK	UK	UK	2023	Short Read	SPAdes (v3.15.5)	Saunders et al., 2024		20.04	*R. pickettii*	*R. pickettii*
